# 

**Published:** 1897-09

**Authors:** 


MARRIAGES.

On Tuesday, August 31, 1897, Dr. Jacob Helmer and Miss Martha E. Shoemaker were married at Syracuse, N. Y. Dr. and Mrs. Helmer will be at home at No. 311 Spruce Street, Scranton, Pa., after October 1st.

### SOCIETY PROCEEDINGS.

### COMING MEETINGS.

THE PENNSYLVANIA STATE VETERINARY MEDICAL ASSOCIATION will convene at Franklin, Pa., September 21, 1897.

Mayor Jobson, of Franklin, Vice-President of the Association, will welcome the members and delegates, and confer upon them the freedom of the city.

Every active veterinarian in Pennsylvania should attend this meeting.

All should come and hear President Rayner's address, with references to the early history of the profession in the Keystone State.

State Veterinarian Pearson will speak upon the future work of the profession, and how far legislation may conserve these interests.

Secretary Rhoads will, in his report, outline the work done, the duties of the members, and how far they will contribute to the success of his administration by their aid.

We have heard the name of Dr. Francis Bridge suggested for Treasurer, and a first-class officer he would make, to succeed our late Treasurer, Dr. John R. Hart.

Reports of interesting and instructive cases will be afforded those present by Drs. Noack, Harger, and Allen.

Papers of great practical interest by Drs. Jobson, McCray, Pearson, and Hoskins will furnish material for much thought.

There are a large number of good veterinarians in the northwest, central west, and southern tier of counties whose names should be enrolled as members, and they should be at Franklin on the 21st to aid in the work for a stronger organization.

Every arrangement has been completed by the local committee: a suitable hall, special hotel accommodations, visits to the great breeding establishments near Franklin—everything to make you welcome and add to your enjoyment.

These semi-annual meetings afford us the only opportunity of visiting sections of our State where we can enlist strong sectional support and enlarge our membership; for this reason, every old member should be at his post and contribute his share of the work.

There are not enough members yet who never miss a meeting. This number is composed of the busiest men in the profession, and you should be among them. It is a roll of honor.

"Why we visit Franklin?" may interest and afford you some food for afterthought and consideration.

You want to go to Franklin and get acquainted with the new officers. You want to be in touch with Secretaries Rhoads and Helmer.

The JOURNAL can only tell you in October a brief part of the work at Franklin. You want to hear it all, and add your mite.

THE NEW YORK STATE VETERINARY MEDICAL ASSOCIATION will convene its eighth annual meeting September 14th and 15th. Are you going? Syracuse, N. Y., has been selected.

The programme is more than interesting, and contains names that attract by their reputation.

Dr. Roscoe R. Bell will deliver an interesting paper on "Infectious Catarrhal Fever of Horses."

Professor James Law will suggest how to best prepare contagious products for shipment.

Dr. W. L. Williams, whose frequent contributions to the JOURNAL are always interesting, will read a paper on "Caudal Surgery."

The rest of the programme is equally as good, and all those who attend will find themselves well entertained.

It is incumbent on every member to give voice to his opinions in the revision of the By-laws, especially those touching the conditions for membership and the increasing of the annual dues.

Two questions of interest are to be presented for discussion: "That the Department of Agriculture Should Have Full Control of the Suppression and Eradication of Tuberculosis and Glanders," and "Would it be Practical and Economical to Create and Maintain the Office of State Veterinarian?" Such questions are vital, and should have the careful consideration and expression of every New York Veterinarian.

The excuse "too busy" is not worth while considering when it comes to association meetings. The most interested are the busiest practitioners, and they are the ones who always find time to attend.

Secretary Morris has labored unceasingly to crown the meeting with success, and he should be aided by every member of the Association. Your interest can be best assured by your presence at the meeting; your aid can be best volunteered by your contributions, however small, to its success.

THE KEYSTONE VETERINARY MEDICAL ASSOCIATION, of Philadelphia, will inaugurate its winter series of meetings on the evening of September 14, 1897, at the hall, northwest corner of Broad and Filbert Streets.

It is expected that Dr. James Johnston will address the meeting on the subject of laws for the compulsory examination of food-products, and what are found as evidences of its need by meat-inspectors.

Dr. James T. McAnulty will briefly outline the future work of the school of farriery.

Dr. Pearson will give a five minutes' talk on the colleges and their sessions for 1897.

Report from the delegates to Nashville will be presented and be fruitful of much interest.

What we hope for at Franklin, Pa., a week later, will be of additional importance. The selection of delegates for this meeting will be decided.

Many other matters and plans will be considered, all of interest to every local veterinarian.

*You* will be welcomed, whether a member or not.

U. S. V. M. A. With Boston, Mass., Helena, Mont., and Omaha, Neb., in the field for the meeting of 1898, surely there will be rivalry enough to make the race interesting and entertaining; surely the most sanguine members of ten years ago could hardly have hoped for such progressive changes in the National Association. So much for good officers. What will the next ten years' progress exhibit? Who will prophesy?

### PERSONALS.

Dr. John R. Mohler, of Philadelphia, was transferred on April 1st from El Paso, Texas, to San Diego, Cal., for the inspection of Mexican cattle importations.

Dr. J. H. Ferster, of Brooklyn, fills the post of veterinary editor to the *Metropolitan* and *Rural Home*, as well as the United States Government veterinary inspectorship.

Dr. Raymond Barber, of Doylestown, Pa., has succeeded to the practice of Dr. H. E. Wand, of Newtown, Bucks County, Pa.

Dr. H. D. Gill, of New York, is a frequent visitor to Coney Island, and, with a fast trotter on the road, whiles away many enjoyable hours.

Dr. Harry Walters, of Wilkesbarre, Pa., was a recent visitor to Philadelphia and New York.

Dr. Alexander Glass, of Philadelphia, was the captain of a party who spent some ten days in August along the shores of the Chesapeake Bay.

President F. H. Osgood, of the U. S. V. M. A., was a victim of influenza during the month of August.

Dr. Albert H. Sheldon, of Boston, is an ardent canoeist, and longs for his annual pilgrimage to the streams of the "Pine Tree State."

Dr. H. A. Christmann has changed his location from Chestnut Hill to West Philadelphia.

Dr. S. J. J. Harger was a recent visitor to Canada on a professional mission.

Dr. George H. Bailey, of Portland, Maine, finds much pleasure at his stock-farm at Deering, Maine, where his choice selection of trotting stock attracts many visitors.

Dr. Charles P. Lyman, of Harvard Veterinary College, finds the mountain air of East Jaffrey, New Hampshire, peculiarly beneficial to his health.

Dr. Wesley H. Labaw, of Boston, spent his summer holidays at his home near Princeton, N. J.

President Lester H. Howard, of the American Veterinary College Alumni Association, and family, of Boston, are summering at Clinton, Mass.

Dr. Leonard Pearson was a visitor to the New England coast in August.

A cablegram from Berlin, Germany, brings news of the arrest of Dr. G. Leo Hagen Burger, of Brooklyn, N. Y., on the charge of theft of surgical instruments from one of the hospital clinics he was attending. Some months ago Dr. Hagen Burger sold out his hospital and practice in Brooklyn, ostensibly to take up the profession of law; but we are informed that his sojourn in Europe was for the purpose of fitting himself in some specialty of veterinary science, with a view of returning to take up practice again in this country. Happily the doctor was released by the judge who declared he did not think Dr. Hagen Burger intended stealing the instruments.

The JOURNAL is glad to report the return of Dr. Charles T. Goentner, of Bryn Mawr, Pa., to practice, with the promise of a complete recovery from his recent alarming illness.

Dr. J. C. Shaub, of Lancaster, Pa., was a visitor to the JOURNAL office in August. His summer holidays are spent in visiting distant sections of the country by easy jaunts in his carriage.

Dr. G. Howard Davison, of Millbrook, N. Y., is an admirer of the Berkshire breed of swine, and was among the purchasers at a recent sale of high-class stock.

Dr. W. S. Manley, of Arkansas City, has relinquished practice for a period, and will enter one of the veterinary colleges for a post-graduate course.

Dr. J. T. Crosby, M. R. C. V. S., formerly of St. Louis, Mo., has purchased the good-will and practice of Dr. W. S. Manley, of Arkansas City, and moved to that place.

Dr. D. P. Frame, of Colorado Springs, holds the position of food-inspector for that city under appointment by the health department. His duties cover meat, milk, and dairy as well as the inspection of all other goods.

Mr. Harold Sorby, of the Pasteur Vaccine Company, of Chicago, who has accomplished so much for the introduction in America of the products of the parent company of Paris, France, will be a welcome visitor at Nashville.

Dr. Matthew Wilson, Jr., of Mendota, Ills., has been appointed on the Board of Horseshoers by Governor Tanner.

Dr. W. W. Richards, of the class of '97, of Ontario Veterinary College, has been travelling in the Northwest, but has finally located at San Diego, Cal.

Dr. T. D. Hinebauch, of North Dakota, in connection with his trip to Nashville spent some time in the leading Western cities.

Dr. M. W. Tritschler, of Cincinnati, Ohio, was a visitor to the JOURNAL office in August. He was off for a summer vacation.

Mr. John A. Logan, Jr., will manage the harness and saddle classes at the Chicago Horse Show, November 2 to 13, 1897.

Dr. Robert McKibben has finally located at Parkersburg, W. Va. Dr. McKibben is one of the recent graduates from the veterinary department of the University of Pennsylvania.

Dr. L. Kenneth Green, of Booth's Corners, Pa., has been appointed inspector under the Bureau of Animal Industry and assigned to duty at Buffalo, N. Y.

Dr. A. W. Clement, of Baltimore, was a visitor to the New England coast in August, and was the guest of Editor Gill, of New York, on his way to the eastern shore of Massachusetts.

Dr. Frederick H. Osgood has again received the appointment of inspector of animals and provisions for the town of Brookline, Mass.

Dr. James B. Rayner and wife, of West Chester, Pa., spent a portion of August at Atlantic City, N. J., in the hope of restoring Mrs. Rayner's impaired health.

Dr. C. P. Lyman, of East Jaffrey, New Hampshire, was confined to his bed by illness for a period in August.

Dr. John M. Parker, of Haverhill, Mass., recently met with the misfortune of the loss of his esteemed wife.

Dr. E. E. Bittles and wife, of New Castle, Pa., spent five weeks travelling through the West. Visited seventeen different States and Territories, and found it required but very few veterinarians to supply the wants west of the Missouri River. In San Francisco an Italian is the leading veterinarian.

Dr. Frederick H. Osgood was recently elected an associate member of the Association of Military Surgeons of the United States, the first recognition of a veterinarian in that body.

Dr. J. G. Parslow, of Marshalltown, Iowa, fills the post of secretary to the racing association of that town.

Dr. Eugene W. Sullivan is veterinarian to the fire department of the city of Chicago.

